# Supplementary material for: The Tissue Architecture of Oral Squamous Cell Carcinoma Visualized by Staining Patterns of Wheat Germ Agglutinin and Structural Proteins Using Confocal Microscopy
Source: Cells. 2021 Sep 18;10(9):2466. doi: 10.3390/cells10092466 (PMC8465371; doi:10.3390/cells10092466)
Supplement: Supplementary file 1 [file cells-10-02466-s001.zip › cells-1340904-supplementary.pdf]

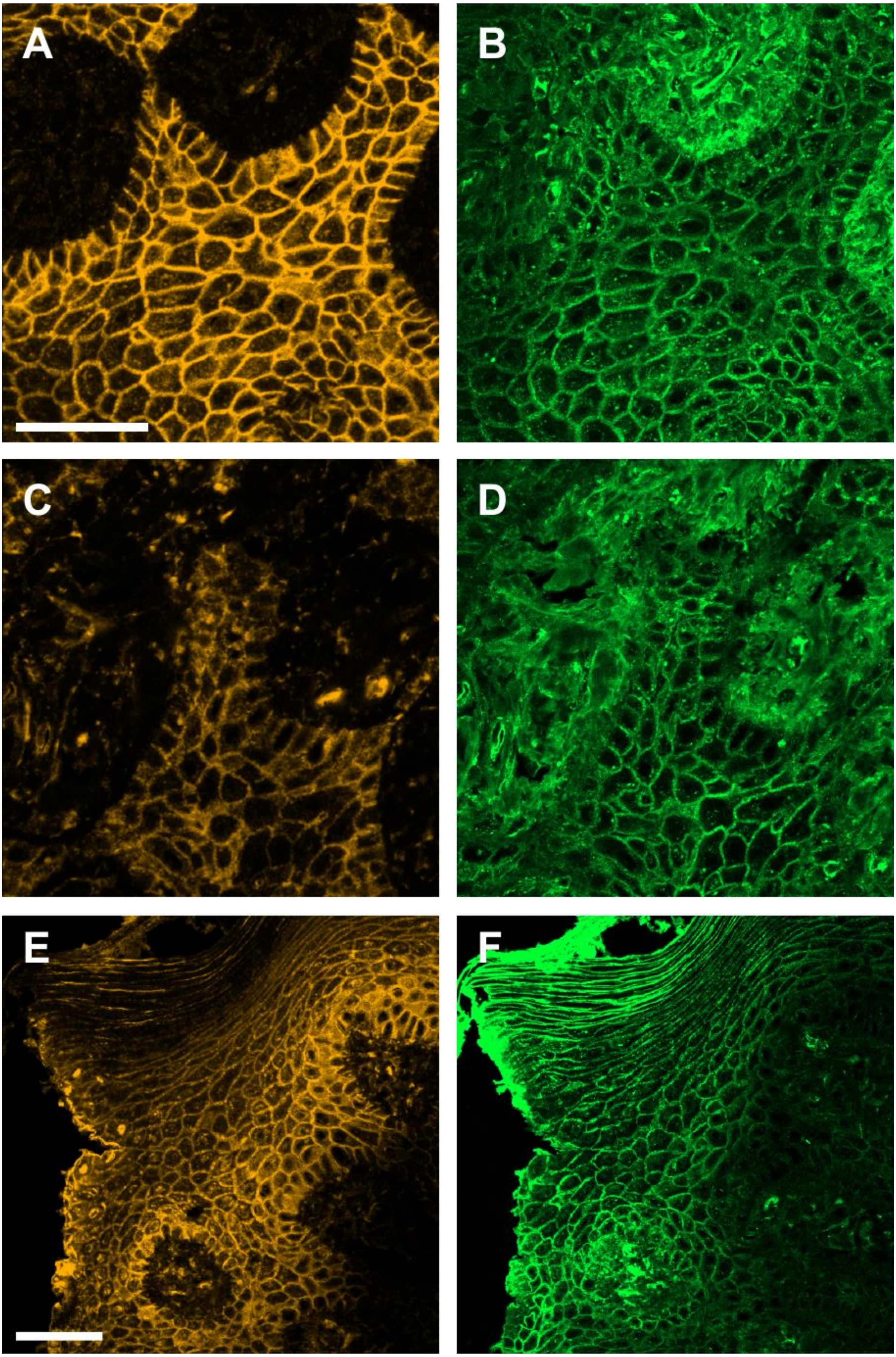
A
B
C
D
E
F

Figure S1. E-cadherin, β-actin and syndecan-1 staining patterns (yellow) in normal oral mucosa; 40× objective. (A) and (B): same field with E-cadherin and WGA (green) staining, respectively. (C,D): same field with β-actin and WGA staining, respectively. (E,F): same field with syndecan-1 and WGA staining, respectively. Scale bars: (A–D) 20 μm; (E,F) 20 μm.
